# 3D dynamic fashion design development using digital technology and its potential in online platforms

**DOI:** 10.1186/s40691-021-00286-1

**Published:** 2022-03-11

**Authors:** Kyung-Hee Choi

**Affiliations:** School of Global Fashion Business, Hansung University, 710 Research building, 116 Samseongyoro-16gil, Seoul, 02876 South Korea

**Keywords:** 3D dynamic fashion garment, Digital technology, 3D virtual simulation system, Online platform, Transformation, Customization, Sustainability

## Abstract

The purpose of this study is to develop 3D dynamic fashion garments with changeable styles, colors and textile patterns, especially using a 3D virtual simulation system, and to examine their potential possibilities in online fashion platforms. For designing and developing 3D dynamic fashion garments, CLO3D and Aftereffects were used with a design collaboration between a fashion designer and a motion graphic artist group. Ten samples of a ready-to-wear collection were developed to visualize aesthetic and technological explorations of 3D dynamic fashion garments, composed of short videos. This study also evaluated the potential of 3D dynamic fashion garments along with 3D virtual simulation systems through focus group interview with professional fashion designers and digital experts, which indicated social and industrial possibilities toward them. This survey showed a list of findings in terms of current status of 3D virtual simulation systems and their impact on companies, discourses on the uncanny valley surrounding avatars, changes in fashion design process derived from 3D virtual simulation systems, co-design and customization in online platforms, and future prospects of 3D virtual garments in the fashion and gaming industries. Finally, this study led to discussions on future possible digital fashion design with several implications, based on design criteria, including digital technology, dynamic range, wearability, expressivity, interactivity, sustainability, and context.

**Supplementary Information:**

The online version contains supplementary material available at 10.1186/s40691-021-00286-1.

## Introduction

The introduction of digital technologies has challenged us to develop new ways of thinking and working to augment the innovative potential of fashion design and change design practices. The increasing prevalence of digital tools in our everyday lives can provide fashion garments with greater flexibility and variability, and their elements can be changed into completely different substances. Quinn (2012) noted that fashion materials in the future will be more fluid than fixed, responding, changing and adapting to sets of preprogrammed parameters. When we consider the nature of changes that are inevitable for the fashion industry due to consumers’ demand, the limitless and dynamic possibilities of fashion garments, interwoven with digital technology, look fascinating and show great promise for the fashion design process as well as the fashion industry in general.

Moreover, COVID-19 has moved up the digitalization of the fashion market and the rise of the virtual world. As COVID-19 locked down many countries worldwide and prevented physical contact between designers and manufacturers, the crisis not only forced fashion industries to inevitably turn to digital and virtual fashion but also provided an opportunity to redefine business models toward more sustainable and digital innovation. Matthew Drinkwater, head of the Fashion Innovation Agency (FIA), noted that COVID-19 is forcing brands to engage and experiment with immersive technologies (Roberts-Islam, 2020). He claimed that the requirement to integrate all forms of digitization from the supply chain and creation to showcasing and retailing is forcing every brand to embrace the technologies that empower this innovation.

In this study, dynamic fashion design is defined as fashion garments with transformable styles and animated colors or textile patterns that visibly change from the garments’ underlying colors or patterns, and even details, to others and then return to the initial condition after a period of time. This demonstrates the potential of transformable digital expressions and aesthetics, as well as technologies programmed to this effect.

As digital technology, including computer graphic software, has become available for fashion and textile designers, digital aesthetics have provided inspirations for new design ideas and visual expressions. 3D virtual simulation systems such as CLO3D and Marvelous Designer of CLO Virtual Fashion and DC Suite of Digital Clothing Center are used by fashion and game designers alike, and many industrial solutions for garment product development, including Browzwear V-Stitcher, Optitex PDS, and Lectra Modaris 3D now feature 3D visualization as well as digital pattern construction (Makryniotis, 2018). In this sense, to display customized styles and various transformable colors and graphics in fashion design, we can create 3D dynamic fashion garments, using specific computer programs by encoding digital-based style, color and pattern information with computational hardware.

Current studies on textiles and garments integrated with technology tend to involve not only technical dimensions but also emphasize their potential for aesthetic expression and playful experimentation. For example, studies on dynamic textiles and garments have been conducted on high-performance conductive materials and thermochromic inks that change colors, textures and forms within textiles (Berzowska, 2005; Orth, 2004; Post et al., 2000; Robertson et al., 2008; Worbin, 2010). Custom-made personal objects, including dynamic textiles, can communicate and interact wirelessly with other technology systems using optical fibers and light-emitting diodes (LEDs), as seen in Philips’ emotional dress in 2006, Hussein Chalayan’s LED dress in 2008, and CuteCircuit’s Galaxy dress in 2008. In addition, digitalized dynamic fashion garments that combine images from computer graphic software with portable hardware such as a mobile phone open up the possibility of creating still and moving displays of graphic patterns that are more personalized and varied than tangible patterns, as many examples, such as CuteCircuit and Shiftwear, have shown. Added to this were the formidable Internet and the growth of social networks, which have provided an indispensable creative platform for users and designers to communicate and collaborate on a variety of design ideas.

Recently, sociocultural directions toward digital garments and technology adoption have also been emphasized in studies (Buechley et al., 2008; Devendorf et al., 2016; Dunne et al., 2014; Farren & Hutchison, 2004; Mackey et al., 2017, 2019; Tomico et al., 2017). Makryniotis (2018) noted that digital dress has the potential to become a strong link between the electronic entertainment world and the fashion world, both of which incorporate three aspects of identity, representation and commerce to certain degrees. In particular, digital garments lend themselves to experimentation with fashion design and even customers’ body images, inhabiting avatars in virtual social environments. Furthermore, the emergence of the web has produced a sense of space that is not only virtual in terms of its intangibility but also simultaneously discursive in terms of “cybernetic space” (Mitra, 2003, p.4), allowing individuals and institutions to transform both the real and the virtual as they influence each other through a networked device. In this sense, practices and discussions of industrial fashion designers should be considered: How they develop their design practices using digital technology, and what kinds of discourses they put on 3D dynamic fashion design for online platforms.

In this study, the author intends to develop 3D dynamic fashion garments with transformable styles, colors and textile patterns for online platforms, using a 3D virtual simulation system, and to explore their potential possibilities by exploring the reactions of fashion designers and digital experts. Therefore, as Makryniotis (2018) considered the components of digital dress as three aspects of the programmatic, the visual, and the social, this study includes two foci as follows.

From an aesthetic and technological aspect, this study aims to create a 3D dynamic fashion design with virtual avatars using CLO3D and Aftereffects, with a design collaboration between a fashion designer and a motion graphic artist group. This is a follow-up study (Choi, 2019a, b), which presented a dynamic fashion design by creating a static fashion illustration, embedding moving graphical patterns for aesthetic, expressive or communicative purposes. In another aspect, this study focuses on investigating social reactions and commercial possibilities toward 3D dynamic fashion garments, alongside the current state of a 3D virtual simulation system by performing a focus group interview (FGI). For this study, the following contents were organized and addressed.

1. To identify the concept of dynamic fashion design and its effects in virtual spaces through prior cases.
2. To explore 3D virtual simulation system and its prospects in the online fashion industry.
3. To examine sociocultural dimensions of virtual fashion garments in online platforms.
4. To develop 3D dynamic fashion design and investigate its technical procedure.
5. To perform a FGI with digital fashion designers and discuss the potential of 3D dynamic fashion design in online platforms.

## Literature review

### The concept of dynamic fashion design and its effects in virtual spaces through prior cases

Worbin (2010) stated that a dynamic color is a color that temporarily disappears to reveal a fabric’s underlying color or display another printed color, and dynamic textile patterns show an inherent quality to change expression during use, from one to another, or several other expressions. Mackey et al. (2017) considered dynamic fabric to be a textile with computational input that enables changes to its visual appearance for aesthetic, communicative and expressive purposes. Kleinberger and Panjwani (2018) regarded digital dresses as dynamic displays that use either color-changing pigments, direct lighting sources (such as LEDs), or projections to accomplish changing visualization on the surface of the garment. Key among these is the notion of one garment functioning as multiple garments (Devendorf et al., 2016; Dunne, 2010; Mackey et al., 2017).

In this study, the author defines 3D dynamic fashion design as digital fashion garments with transformable styles and animated colors or textile patterns that visibly change from the garments’ underlying colors or patterns, and even details to others, and then return to the initial condition after a period of time. It is simulated and animated with 3D moving visualizations in a virtual space, and it demonstrates the potential of transformable digital expressions and aesthetics, as well as technologies programmed to this effect.

In fact, transformable clothing can be referred to the garments, which offer multiple functional and/or aesthetic alternative styles (Farrer, 2011; Koo et al., 2014; Rahman & Gong, 2016). In addition, transformable garments do not only include garments transformed into different styles with changing their forms or silhouettes, but also ones transformed with surface decorations and embellishments, and even smart clothing (Rahman & Gong, 2016). In this regard, dynamic fashion in this study has the same meaning as transformable fashion.

The term, “dynamic” has been usually introduced in textile design, especially digital or smart fabrics, as a meaning to change expression during use, from one to another or several other expressions, as opposed to fixed and static (Berzowska, 2005; Orth, 2004; Post et al., 2000; Robertson et al., 2008; Worbin, 2010). Moreover, several studies about digital fashion, focusing on dynamic surface design have also used the term “dynamic” (Choi, 2019a, b; Kleinberger & Panjwani, 2018; Mackey et al., 2017, 2019). Therefore, although dynamic fashion in this study means a sort of transformable fashion, the author would like to keep the term, “dynamic” for this study because this study started from digitalized dynamic textiles and garments with computational input.

To date, there have been already a disruptive leap of dynamic textiles and fashion garments, whether they are tangible or virtual, out of the realm of human–computer interaction (HCI) research, catwalk shows, performance art, and industry. As Worbin (2010) classified technological principles of dynamic fabrics with or without computational technology into thermochromism (TC) by heat, photochromism (PC) by exposure to ultraviolet light, and electroluminescence (EL) by light or by optical fibers or luminescent materials, there have been not so few physical examples of dynamic textiles and garments (Choi, 2019a, b).

Given the aesthetic and expressive effect of dynamic fashion design in virtual space, we can consider screen-based (i.e., video-mapping projections) dynamic fashion garments, which are virtual or virtual-physical with certain digital applications or computer software. Basically, “virtual” means “digital” being on or simulated on a computer or computer network (Merriam-Webster, n.d.; Mitra, 2003, p.3). Within the last five years, dynamic fabrics which combine virtual-physical means as in augmented or mixed-reality technologies have appeared in fashion (Farren & Hutchison, 2004; Mackey, et al., 2017). Thus, speculating on various cases of fashion design, which merges virtual dynamic fabric-like surfaces with physical garments, the author examined and analyzed prior cases of virtual and virtual-physical dynamic fashion garments and their effects for aesthetic and technological inspirations of this study.

The criteria to categorize the prior cases covered a specific list of the following characteristics: digital technology, dynamic range, dynamic elements, interactivity, expressivity, sustainability, and context. The categorization was based on previous studies of digital fashion or dynamic garments: Chun (2011) characterized digital art and digital fashion as having “perfect duplicability”, “interactivity”, “networkability”, “variability” and “compositeness”. Clarke and Harris (2012) stated that digital media provide imagined, screen-based scenarios, incorporating imagery that is abstract, hyperreal, macro, time-based, self-generating and fast evolving. Kleinberger and Panjwani (2018) categorized previous cases of dynamic digital displays by display technology, wearability, interactivity, brightness and context. Choi (2019a, b) grouped the characteristics of digital dynamic fashion illustrations into “variability”, “composition of different elements”, “multiple duplicability”, “interactivity”, “hyperreality”, “sustainability”, and “cyclic iterativeness”. Since the definition itself of dynamic fashion design includes the features of variability, duplicability, compositeness, and iterativeness, the author analyzed the prior examples of virtual (or virtual-physical) dynamic fashion design by the criteria of wearability, expressivity, interactivity, and sustainability and added the information of digital technology used, dynamic range, and context (Table 1).

**Table 1:** Prior cases of virtual or virtual-physical dynamic fashion design and its effects

No	Title	Year	Digital tech	Dynamic range	Wearability	Expressivity	Interactivity	Sustainability	Context
1	Jane Harris’ s ‘Potential Beauty’	2002–3	3D CG modeling with motion capture	Virtual	On screen only	Motion of form and textile (twisting, folds, draping)	No	Yes	Video artwork
2	Viktor & Rolf’s ‘Long Live the Immaterial’	2002–3	Video-mapping with a blue-screen projection	Virtual-physical	Fully portable	Display of nature and city scape	No	Yes	Catwalk show
3	UVA & Hamish Morrow’s ‘Beauty of Technology’	S/S 2004	Video-mapping projection with 3D flashes	Virtual	Fully portable	Display of digital print shadows	No	Yes	Catwalk show
4	Hussein Chalayan’s ‘Airborne’	A/W 2007–8	15,000 LEDs & video screen	Virtual-physical	Fully portable	Animated prints	No	Yes	Catwalk show
5	Studio XO & Nancy Tilbury’s ‘Digital Skins’	2011	Video projection onto skin (with a sensor)	Virtual-physical	Limited range	Color and pattern change on skins	No	Yes	Design experiment
6	Frank Sorbier’s dress	2012	Video projection (front)	Virtual-physical	Immobile	Color and pattern change on a garment	No	Yes	Haute couture
7	CuteCircuit’s ‘Infini T-Shirt’	2012	Mobile applications & small, battery-powered computer	Virtual-physical	Fully portable	Display of images, animations, tweets photos & music play	Yes	Yes	Commercial product
8	Marga Weinmans	2013	AR technologies with mobile applications	Virtual-physical	Limited range	Moving patterns	Yes	Yes	Catwalk show
9	Jennifer Lopez’s ‘Feel the Light’ dress	2015	Video-mapping projection	Virtual-physical	Immobile	Display of animated film	No	Yes	Show costume
10	Ece Ozalp’s What is Real?	2015	Front/side projection	Virtual-physical	Immobile	Display of pattern changes	No	Yes	Haute couture
11	Studio Performa’s ‘DROMe’	2015	Front/side projection	Virtual-physical	Immobile	Display of pattern changes	Yes: sounds	Yes	Design experiment
12	Kailu Guan	2016	AR technologies with mobile applications	Virtual-physical	Limited range	Moving patterns	Yes	Yes	Catwalk show
13	Mirror Mirror	2016	Front projection by a mirror display	Virtual-physical	Limited range	A virtual fitting system	Yes: motion and action	Yes	HCI research
14	Ishikawa Watanabe Laboratory system	2016	Front projection by an invisible IR ink for the projected images	Virtual-physical	Limited range	Display of pattern changes	Yes: motion	Yes	HCI research
15	Swiftwear corp’s ‘Swiftwear sneakers’	2015–17	Mobile applications & E-paper screen	Virtual-physical	Fully portable	Display of animated designs & customized images	Yes: mobile app	Yes	Commercial product
16	Wu et al.’s ‘Transforming dress’	2013	DC Suites, Maya & Aftereffects	Virtual	On screen only	Display of form, color and texture changes	No	Yes	HCI research
17	Mackey et al.’s ‘Greenscreen Dress’	2017	Mobile screen & Chroma-keying mobile app	Virtual	On screen only	Fabric-like surfaces with colors and pattern changes	Yes: mobile app	Yes	HCI research
18	Kleinberger & Panjwani’s ‘Enchanted Wearable system’	2018	Video-mapping projection	Virtual-physical	Fully portable	Moving patterns	Yes	Yes	HCI research
19	Mackey et al.’s ‘Phem’	2019	Mobile screen & Chroma-keying mobile app	Virtual-physical	On screen only	Digital shimmers with color and pattern changes	Yes: mobile app	Yes	HCI research
20	Choi’s ‘Psychedelia’	2019	Adobe graphic programs	Virtual	On screen only	Fashion illustrations with color and pattern changes on garments	No	Yes	Video artwork

The case review results of the prior examples of dynamic fashion design are presented in Table 1, although there are more cases. From the survey, the author determined that digital technologies used to create dynamic fashion design in virtual spaces were mostly video-mapping projection from the front, side, or rear projection, or using screen-based technology, which were integrated into mobile applications, AR (augmented reality) technologies, and computer graphic software programs, such as chroma-keying technique, 3D CGI (computer-generated imagery) and 3D flashes. As the dynamic range included virtual and virtual-physical ranges, wearability ranged from on screen only, immobile, limited range, to fully portable. In terms of expressivity, most prior instances showed dynamic moving displays in colors and graphics, focusing on canvas-based surface design, although Jane Harris’s Potential Beauty and Wu et al.’s Transforming Dress expressed motion of forms, as well as textiles, and Mirror Mirror showed a virtual fitting system. Some examples presented interactivity: Mirror Mirror and the Ishikawa Watanabe Laboratory system reacted to wearer’s motions, Studio Performa’s DROMe to sounds, CuteCircuit’s Infini T-Shirt, Swiftwear corp’s Swiftwear sneakers, Mackey et al.’s Greenscreen Dress and Phem to mobile applications, and Marga Weinmans and Kailu Guan’s shows to AR technologies with mobile applications. All the cases showed sustainable possibilities with multiple transformations. The prior cases included various contexts, such as catwalk shows, haute couture, show costumes, commercial products, video artworks, and academic researches.

In particular, Mackey et al.’s (2017, 2019) two studies gave much inspiration to this study. Mackey et al.’s (2017) Greenscreen Dress is a system for exploiting green garments using a chroma-keying mobile application. In this study, they incorporated green color into a wardrobe on a daily basis and captured digital expressions of garments inscribed with changing digital content. Along with an expressive approach to wearable technology, rather than technological functions, Mackey et al. (2019) also introduced a fashion brand, Phem. Presenting fashion garments constructed with surface-changing, animated images by way of augmented reality with chroma-keying applications, they explored how digital shimmers and physical materials could intertwine through a fashion film.

Wu et al.’s (2013) Transforming Dress has similarities to this study in showing transformation factors, such as shape, color and textile of the dress, using one of 3D virtual simulation systems, DC Suite. However, it focused on transformable effects between two dresses, rather than multiple changes, and it discussed neither changes of dynamic textile patterns within dresses, nor any evaluation of them. Other than that, no prior cases of dynamic fashion design, using CLO3D have been found yet, despite many work cases of CLO3D in social network spaces. HADEEART’s (n.d.) Transformer Dress as a similar case shows a full outfit that can be detachable and worn separately, rather than a dynamic dress with transformable design elements.

At the center of these cases, there is a desire to move toward wearable applications for flexible, fabric-like surfaces that can be visually transformed through computation, which contributes to the interaction between garments and wearers and emphasizes sustainability with the variability of dynamic fashion in daily life. Given the review result of the prior cases, dynamic fashion garments need to be developed toward fully multiple transformation in form and style as much as surface transformations, which can be also equal to the textual expressions of physical fabrics rather than flat screen-based ones. When discussing the potential of dynamic fashion design at the end of this study, the author will return to this aforementioned categorization.

### 3D virtual simulation system and its usage in the online fashion industry

Since the mid-1990s, computer-aided design and digital media, which can be used with fashion garments, have brought about further refinements of high fashion, entertainment-related animated garments and even commercial products. As the use of imaging software such as Photoshop, Illustrator and Aftereffects has been common in various sectors with the influence of the Internet, 2D, 3D, or 4D digital tools have also enabled the forms and motions of garments, as well as their surfaces and textures to present new digital aesthetics (Clarke & Harris, 2012). Fashion narratives in online space have not been less significant means than in offline space, as they enhance the technological and aesthetic effects of tangible garments rather than just their alternative entities. As online shopping has been successfully competing with offline markets and consumers have been spending more time in online spaces, fashion industries still need to explore the potential of what online fashion offers. In addition, fashion film, a commonly used artifact of the fashion field, has started to digitize human form and movement, using motion capture as well as the aesthetic and expressive aspects of fashion garments to hold space for design inquiry.

With moving-image narratives to describe fabrics in relation to the body, as exemplified in Jane Harris’s Potential Beauty, the newly emerging methods of computer graphics, visualizing textiles and garments can create aesthetic concerns pertaining to animated and real-time forms. In particular, Harris represented subtle characteristics inherent in textiles and garments, such as texture, touch, and handling, including the wrinkling, creasing and folding of cloth, which can make designers and artists overcome the limits of screen-based dynamic fashion garments, such as lack of textual expressions, as mentioned above. Thus, such new digital imaging media led to the development of a variety of 3D computer simulation and animating software programs, taking the physical into the virtual in various online areas.

3D virtual simulation systems have constantly evolved since the advent of 3D graphics can now support these changes. Currently, 3D virtual fashion CAD systems include Browzwear’s V-Stitcher, Optitex’s 3D Suite, Lectra Modaris 3D, Human Solutions’ Vidya, Techno A’s i-Designer, CLO Virtual Fashion’s CLO3D and Marvelous Designer, and Physan’s DC Suite. 3D virtual clothing systems can not only visualize a real-time interaction between 3D simulations and 2D pattern constructions of virtual clothing, but can also embody elaborate materials similar to real garments. Thus, 3D simulation systems contribute to effectively reducing the time and cost of the development process through pre-fitted sampling of a variety of virtual clothing. According to Makryniotis (2018), software programs such as CLO3D, Marvelous Designer, and DC Suite are used by fashion and game designers alike, and there are emerging technologies that facilitate the incorporation of fashion expertise in games, as well as of 3D graphics in fashion. In addition, Virtual fitting mirrors in stores and virtual fitting rooms on e-commerce platforms make use of similar real-time technologies. Therefore, 3D virtual clothing systems have successfully overcome technological restrictions existing in 3D modeling and animation, and they have been expanding their ranges toward virtual game animations, films, advertisements, and even sports, in addition to fashion-related fields.

Of late, there have been a variety of examples of digital technology integrated into virtual fashion, such as digital fashion shows, mobile fashion contents, AR fashion, digital signage, digital installations, online fashion magazines, and look books. Fashion products and retail spaces with digital identities and digital prototyping now transform our environments, and brands increasingly provide opportunities to sell digitally rendered garments to consumers as they become familiar and comfortable with this technology. In autumn 2020, Tommy Hilfiger launched a collection designed and developed using 3D technology with products modeled on virtual avatars, Undercover by Jun Takahashi presented a look book of 3D-rendered images for the S/S 2021 collection, and Balmain showed virtual showroom avatars by integrating VR (virtual reality) technology with fashion story telling. In the 2021 Digital Fashion Week, a brand, SUNNEI Canvas, presented the brand into a new space that merges reality with the virtual world by 3D engineering and a refined customization technology. The brand showed a good example of dynamic fashion in that features such as shapes, fits and fabrics on animated avatars can be modified digitally, while on screen, thus changing the appearance of clothing and accessories according to the visitors’ choices.

5G enables high-quality mobile access, opening up the live reach of social video platforms such as TikTok, streamlining the connection between platforms, communities and influencers and fueling influential product tie-ups. Louis Vuitton's tie-up with League of Legends led to LVMH virtual skins, as well as a product capsule. An online retailer, Yoox, launched its application where shoppers can create, style and share a personalized digital avatar by taking a selfie. The Gucci group set up a Gucci application, where users can download wallpapers, take pictures with stickers and motifs of the house, use AR to decorate spaces and virtually try on their items. In addition, some popular 3D virtual influencers have emerged, favoring online social media platforms, such as Instagram, Facebook, and YouTube. Thus far, as 3D virtual simulation systems and digital imaging media give numerous opportunities to the current fashion industry in online space, as mentioned above, they will be able to provide useful technical devices to develop 3D dynamic fashion garments and explore their effects in the online fashion industry.

### Sociocultural dimensions of 3D virtual garments on online platforms

As 3D virtual garments have provided us with solutions for wearable technologies, new considerations arise for how to balance HCI with aesthetic resonance, social adoption and cultural relevance for the user. Tomico et al. (2017) noted that when technologies are placed on bodies, they participate in informing the aesthetic and communicative layer of fashion that comes to constitute the social self. For fashion designers, virtual garments need to be considered in the realms of embodiment, daily life and sociocultural users, as much as physical garments are.

Digital dresses within a computer game world are obviously related to the player’s identity through the embodiment of the avatar. Body image is defined as a person’s attitude toward the physical self, including thoughts (cognition), feelings (affect), and behavioral evaluations (Thompson & Smolak, 2001), and feedback from others’ perception of oneself (Cash, 2012; Cash & Pruzinsky, 2004; Thompson et al., 1999). Makryniotis (2018) stated the phenomenon that key aspects of personal identity are partly communicated through clothing in the social world and is the same for game characters and virtual world avatars. According to him, the bodies and attires of the characters largely contribute to the signs communicated in multiplayer games and virtual worlds in a kind of social performance, which is largely strategic and expressive. Moreover, the virtual aspect of digital garments as a vehicle of commerce has the potential to become a strong link between the electronic entertainment world and the fashion world with the personal and interpersonal effects of clothing.

Thus far, there have been several prior studies on HCI and the sociocultural aspects of wearable technologies (Buechley et al., 2008; Devendorf et al., 2016; Dunne et al., 2014; Farren & Hutchison, 2004; Joseph et al., 2017; Mackey et al., 2017, 2019; McCann, 2016; Tomico et al., 2017). Joseph et al. (2017) introduced new approaches to the development of soft wearable technologies based on embodied imagination, which integrated the body into the design process in an open-ended way. McCann (2016) explored the necessity of the co-design process to evolve from designing for users to making the persons involved active contributors, which shows the iterative design process. Mackey et al. (2017) focused on the importance of considering sociocultural implications of identity and audiences as well as changes in daily clothing practices and interactions when designing wearable technologies by introducing an auto-ethnographic study. Mackey et al. (2019) also provided garments with animated fabrics by AR with a social meaning in an everyday fashion context, using a fashion film, beyond just entertainment or artistic contexts. The dynamic garments in the study not only represented the expressivity of the digital aspect drawn from cultural references but also negotiated a person’s identity in society, as did the tangible garments.

As the Internet and mobile technology have become ubiquitous in people’s daily lives, Lau and Lee (2019) found more interactive experiences for apparel retailing were created by enhancing consumers’ hedonic shopping experience in stereoscopic VR shopping. Moreover, Park (2018) also found that emotional responses toward viewing their own virtual body and willingness to take part in future virtual experiences were significantly influenced by users’ self-esteem. In addition to the personal gains among users, VR and AR effectiveness can bring many benefits to the fashion industry and the consumer marketplace. For instance, Wang and Chen (2019) found that visual product placement in VR videos had a positive impact on dialogic engagement, which in turn was positively associated with a co-creating experience. Kim et al. (2020) suggested that fashion companies should develop and utilize AR content as a means to stimulate a positive response through curiosity.

Meanwhile, as a 3D virtual simulation system has the potential to save time and labor in the apparel prototype development process, there have been studies on the efficiency of a 3D garment simulation as a substitute for a physical prototype. Prior studies on 3D virtual garment simulation have focused on physical measures of accuracy (Ancutiene et al., 2013) or on end user evaluations and purchase decisions (Kim & Labat, 2012). Porterfield and Lamar (2017) explored the extent to which a 3D virtual garment simulation provided functional realism, that is, the user’s ability to make intent to purchase decisions using a garment simulation to enable fitting and fit evaluation within the context of the apparel development process, baselining how the interactive process of garment fitting might be impacted.

Moreover, online social environments are ideal for experimentation with the avatar’s appearance, as they are usually built for communication between users, and that the users have a certain amount of choice concerning the appearance of their avatars. Online worlds constitute a market of identities, and there is an inherent status-oriented socioeconomic system in virtual worlds (Makryniotis, 2018). With the rise of the emerging platform landscape, Gillespie (2018) defined platforms as online sites and services that host, organize, and circulate users’ shared content or social interactions or them and are built on an infrastructure […] for processing data for customer service, advertising, and profit. These online platforms follow a “social media logic” (Scolere, 2019, p.1), based on key principles of programmability, popularity, connectivity, and datafication. Social media platforms encourage users to share information with practicing connectedness and lead creative professionals to examine self-promotion and self-presentation activities, producing and mediating the meaning of shared content.

Considering the current progress of a 3D virtual garment simulation, it would be worth discussing the efficacies of 3D virtual simulation systems and the reactions of 3D dynamic fashion garments for online platforms, among fashion designers and digital experts as creative professionals.

## Methods

### Technical procedure to develop 3D dynamic fashion garments

Fashion garments can be dynamic, no longer static, and the same piece of clothing can fulfill a wide range of multiple looks to match and represent the ever-changing desires of the consumer/wearer. To design and develop 3D dynamic fashion garments, this aesthetic and technological study aims to present the effects and future potential of 3D dynamic fashion design simulated with virtual avatars in an online space through design collaboration between a fashion designer and digital textile designers through online social platforms.

For this, a 3D virtual clothing system, CLO3D and a digital imaging program, Aftereffects were used to create virtual avatars in 3D dynamic fashion garments. In particular, CLO3D allows users to instantly review expansive and versatile changes in fashion design as any modifications to 2D patterns, colors, textures and finishing details are immediately simulated, and it contributes to improve the quality of designs by checking silhouette and fit sooner in the development process (CLO Virtual Fashion, n.d.). Compared with the other 3D virtual simulation systems, the current spread of CLO3D is very rapid in both fashion companies and educational institutions (CLO Virtual Fashion, n.d.; Ju & Jeong, 2016). Furthermore, it was essential to use a video-editing program, such as Aftereffects in order to emphasize the effect of dynamic change in fashion garments.

In addition, an online collaboration was formed with a motion graphic artist group, Protobacillus by a social network system, Tumblr, to create digital textiles with moving graphic imagery, which were mapped into fashion garments. As Wang et al. (2017) mentioned that collaboration between textile and fashion designers is the core of creativity, the combination of two diverse design fields will be able to generate new and innovative outcomes in fashion design development through co-creation for relevant themes.

With a theme of dynamic fashion, the author created a ready-to-wear collection composed of ten designs, which visualize and describe experimental explorations of 3D dynamic fashion garments in a wide area of styles, colors, and textile patterns. Based on changes of minimal silhouettes and basic details, the design concept expressed sustainable, geometric, and futuristic dynamism, especially in colors and textile patterns with realistic textures, not screen-based ones. The design samples included three Animations, three Turntables (video capture), and four V-ray rendered images: Animation mode creates stable and realistic garment animation for multilayered garments, a video capture, Turntable records 3D garments with a circular rotating animation, and a V-ray render can produce more vivid and sophisticated images with clear seamlines and puckering, including various views and video properties in CLO3D.

**Fig. 1:**
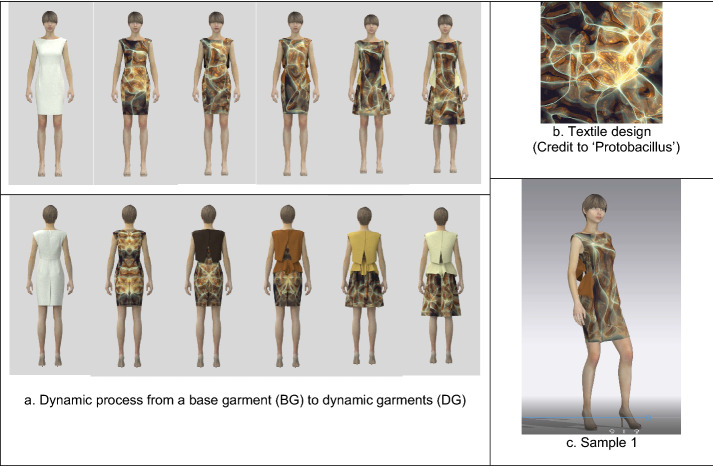
The technical process to develop a 3D dynamic fashion garment (sample 1): **a** The moving images, **b** textile design (GIF), and **c** sample 1 (MP4) are available in Additional file 1

**Fig. 2:**
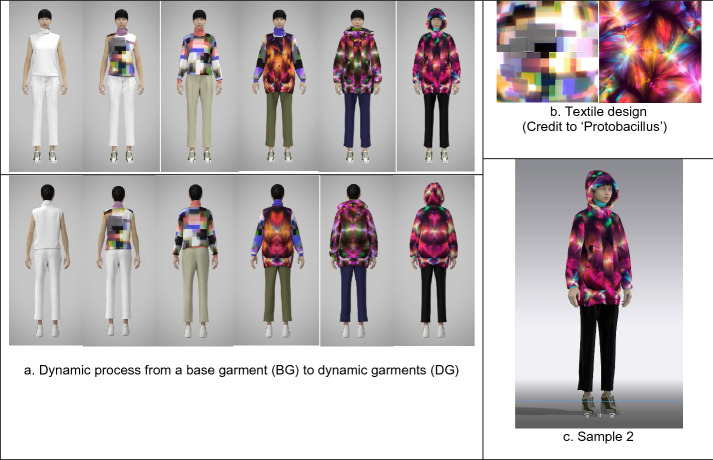
The technical process to develop a 3D dynamic fashion garment (sample 2): **a** The moving images, **b** textile design (GIF), and **c** sample 2 (MP4) are available in Additional file 1

**Fig. 3:**
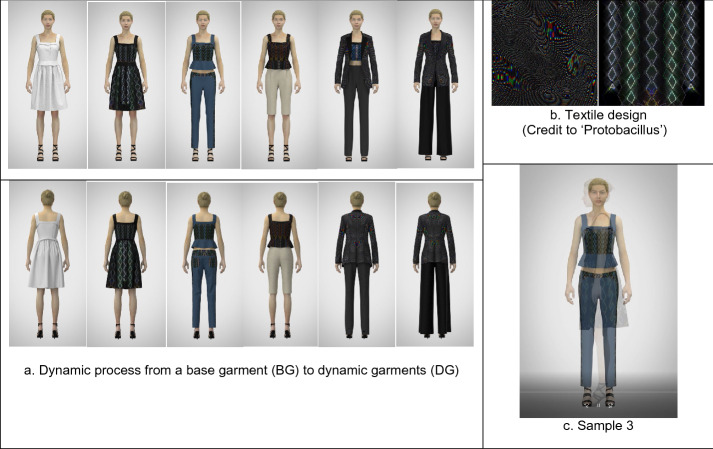
The technical process to develop a 3D dynamic fashion garment (sample 3): **a** The moving images, **b** textile design (GIF), and **c** sample 3 (MP4) are available in Additional file 1

**Fig. 4:**
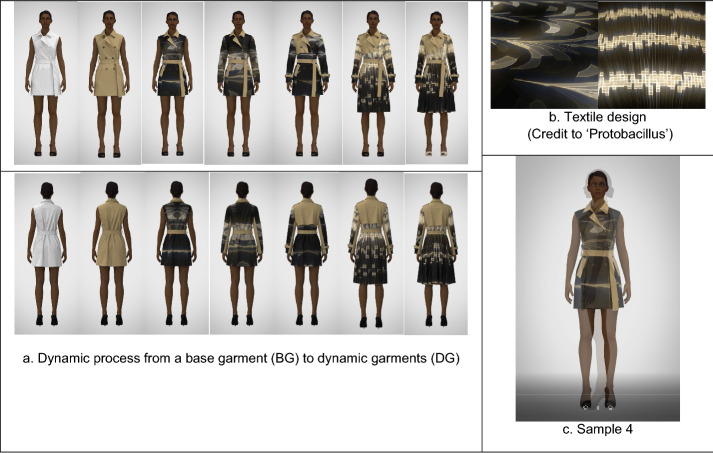
The technical process to develop a 3D dynamic fashion garment (sample 4): **a** The moving images, **b** textile design (GIF), and **c** sample 4 (MP4) are available in Additional file 1

Four samples to represent each type were presented and described in this study to define an overall technical design process (Figs. 1, 2, 3, 4). The major technical procedure for visualizing 3D dynamic garments is as follows:

First, using CLO3D, a base garment with an avatar was designed as a default condition like a canvas, which has items of a basic form and does not include any color, graphical pattern, and texture.

Second, from the base garment, five to six different styles were created by transforming their constructions and details by CLO3D. For example, a basic dress can be changed into a flare dress with different skirt lengths, and several details, such as flaps, belts, and frills can be added to the initial minimal dress.

Third, GIF animations of digital textiles, produced by Protobacillus were taken apart in Photoshop, and fifteen to eighteen fabric images out of all 24 material ones were chosen for a dynamic garment. Each fabric image was mapped in turn within the transformed garment designs above by using graphic tools and texture editing tools of CLO3D.

Fourth, approximately fifteen 3D garments were simulated by CLO3D, and each garment was created into video captures with Animation, Turntable, or V-ray rendering functions of CLO3D.

Finally, all the video captures (approximately fifteen videos) were combined to become a video group by Aftereffects. The video group was edited by several effects of Aftereffects, and eventually became a 3D dynamic fashion garment, which represented transformative forms and details, color changes, and sequential fabric images.

Sample 1 (Fig. 1) exemplifies an animation type of 3D dynamic garments. With a sustainable and futuristic theme of "Guilty plastics", it is transformed from a minimal sheath dress into a longer flared dress with a back detail and a peplum, showing color alterations and animating textile patterns. The technical process of sample 1 is as follows: (1) A base garment (BG) was created as a default condition of 3D dynamic garments (DG) by CLO3D (Fig. 1a). (2) By adding a peplum and back details to the BG, transforming the dress silhouette, and increasing the dress length, five different styles (Fig. 1a) from the BG were simulated by CLO3D. (3) The animating images of a GIF file (Textile design of Fig. 1b) created by Aftereffects, were used as a textile design for the DG. Using Photoshop, the GIF file was taken apart into twenty-four different textile images. Then, fifteen textile images of them were mapped within five different styles in various ways by CLO3D. (4) Each style of five DGs included three textile designs to be transformed, and fifteen garments that change continuously were finally completed. (5) Each of fifteen garments was converted into the animation mode by CLO3D, which represented runway animations with avatars. (6) By combining and editing fifteen animations (AVI files) using Aftereffects, an animation video capture of a 3D dynamic garment was created as a result (Fig. 1c).

Sample 2 (Fig. 2) is a turntable video capture, which represents the process of layering a casual outfit, as well as dynamic mutation, including constructions, details, colors, and graphic patterns. With an avatar rotating circularly, the sleeveless base top is transformed into a long sleeve top with a dynamic graphical pattern, the base pants are altered to show color changes, and a layered vest on the top is changed into a padded, hooded parka with different colors and moving patterns.

Sample 3 (Fig. 3) is a v-ray rendered video capture with sequential four-sided views, which also represents the layering process to wear a formal outfit as well as transformations of items, details, colors, graphics, and even textures. The base garment is a strapped pleat dress, and it is divided into a top and a bottom, which is changed into pants. A jacket mapped with dynamic moiré graphic patterns is layered on the top, shifting with diverse diamond graphical surfaces, and the pants, showing different colors and textures, are continually altered in length and width.

Sample 4 (Fig. 4) is another example of a four-view v-ray rendering video, which shows an avatar in a dynamic trench coat. A colored cotton twill texture is mapped within the base garment, which is continually transformed into different styles as some details, such as sleeves, front and back flaps, a belt, epaulets, and cuff flaps, are sequentially added to it, and the length and width of the trench coat are changeable. Two types of digital textiles are mapped into the garment, creating moving graphical images on its surface.

### A survey of digital users’ reactions to 3D dynamic fashion garments

As digital technology becomes more intimate and ubiquitous in our everyday lives, the sociocultural characteristics of fashion as an everyday lifestyle-driven product category are also considered in online space, which might have an influence on the future fashion industry and fashion design process. This study focuses on digital users’ social perceptions, attitudes, and behaviors toward 3D dynamic fashion garments, as well as the aesthetic or technological dimension to explore their potential in the online fashion industry.

As a qualitative study can provide opportunities for gathering rich insights and understanding unexpected issues through open-ended questions and in-depth interviews, a focus group interview (FGI) was organized as a purposeful sampling method. A focus group is an informal discussion among selected individuals about specific topics (Beck et al., 1986), and the participants are relatively homogeneous, particularly in relation to “prestige” or “status” factors such as occupation, social class, or age (Carey, 1994, p.229). A focus group interview allows the researcher to select subjects whose expert knowledge and experience can offer useful insights into an area of specialized activity (Creswell, 2013). Compared to one-to-one interviews, the focus group is far more appropriate for the generation of new ideas formed within a social context (Breen, 2006). For the FGI, this research was approved by the Korean Public Institutional Review Board (IRB Approval No. P01-202107-23-001) regarding ethical issues.

According to Krueger (2014), although the traditionally recommended size of the focus group within marketing research is ten to twelve people, the ideal size of a focus group for most noncommercial topics is five to eight participants. He also added that large groups more than ten participants would be difficult to control and they might limit each person’s opportunity to share insights and observations. In this regards, participants of the focus group interview for this study were composed of five digital users.

In particular, to evaluate the status of 3D virtual simulation systems and the potential of industries for 3D dynamic fashion garments, this study focused on professional fashion designers’ viewpoints, rather than consumers’ reactions among digital users. Thus, the criteria to select the participants were their occupations and careers, regardless gender and age and also their digital capabilities, including the ability to use CLO3D. More specifically, the participants included two digital fashion designers belonging to the CLO3D team, one fashion designer and digital consultant who launched an individual fashion brand, and two fashion designers of garment vendor companies located in South Korea. In addition, they all had more than three years of work experience in industry, using digital technology, including CLO3D. Although their work periods ranged from three years to fifteen years, it had been three to eight years since the participants started to use 3D virtual simulation systems (Table 2).

**Table 2:** Brief description of the focus group interview participants

Participants	Professional category	Industry type	Work period (years)
Participant 1	Digital fashion designer & consultant	CLO3D team	8
Participant 2	Digital fashion designer & consultant	CLO3D team	3
Participant 3	Fashion designer & digital consultant	An individual designer’s brand & CLO3D team	6
Participant 4	Fashion designer & digital consultant	A garment vendor company & a digital consulting company	15 (5 in digital design)
Participant 5	Fashion designer	A garment vendor company	4

All the participants were digitally savvy, which knowledge on 3D virtual simulation systems and fashion garments, and they were active users around digital fashion in online platforms or online games. For the opening question (Table 3), the participants answered that they were not only interested in news and design works surrounding digital fashion in online platforms, including social media but also often inspired by digital design works posted on social media, such as Instagram or Pinterest. Thus, although the participants’ gender and age vary, their novel insights into the potential of 3D dynamic fashion garments are expected to be discovered.

**Table 3:** Questions of the focus group interview

Opening questions	What experiences have you had of 3D virtual simulation systems or the virtual fashion world? Are you an active user of online platforms about digital fashion?
Introductory questions	How useful are 3D virtual simulation systems, including CLO 3D, in designing or retailing garments under the current states? Especially during COVID-19, is there more significance for 3D virtual garment simulations?
Transition questions	What differences are there between the hands-on design and digital design processes using 3D virtual simulation systems?
Key questions	How do you feel about the ten video animations representing 3D dynamic fashion garments that have been shown? Do you determine any potential from the video of 3D dynamic fashion garments?
Ending questions	Finally, is there anything connected with 3D dynamic fashion garments, virtual simulation systems, or digital fashion, that has not been discussed that you feel strongly about and would like to bring up now?

Considering virtual features of 3D dynamic fashion garments, the FGI was performed by Webex for approximately two hours and recorded under the participants’ consent and transcribed by Clovernaver to ensure accuracy. Before starting the FGI interview, the author provided the participants with an overview of a research topic and informed them of the ground rules of the focus group, including the assurance of confidentiality.

The FGI was structured around a series of open-ended reflection questions (Table 3) to encourage participants to reflect on their digital design practices and thoughts and to obtain unexpected findings. However, it focused on examining designers’ and producers’ viewpoints about the potential of 3D virtual simulation systems and 3D dynamic fashion garments. The questions were also based on the design criteria discussed in the survey on prior cases of dynamic fashion, rather than fit and measurements.

For the reliability of the focus group data, the author and another independent researcher crosschecked the codes of the interview transcription. As Breen (2006) stated, the following two indicators in the codes were considered: (a) the extent to which participants agreed/disagreed on issues (look for issues on which there is general agreement and treat issues on which there is disagreement with caution) and (b) the frequency of participant opinion shift during the discussion (treat higher frequencies of opinion shift with caution).

## Results

This section presents a list of findings related to the potential of 3D dynamic fashion garments from the FGI questions. They include (1) current status of 3D virtual simulation systems and their impact on companies, (2) discourses on the “uncanny valley” surrounding avatars, (3) a fashion design process with 3D virtual simulation systems, (4) 3D dynamic fashion design: co-design and customization in online platforms, and (5) future prospects of 3D virtual garments in the fashion and gaming industries.

### Current status of 3D virtual simulation systems and their impact on companies

For the introductory questions, the participants commonly considered the most significant advantage of 3D virtual simulation systems the immediacy of 3D garment simulations, which enables garment designers to design their works and modify them easily at one time. The participants also noted that 3D virtual simulation systems could contribute not only to saving time and cost but also to protecting the environment because it allows designers to skip a toiling stage. In fact, those advantages of 3D virtual simulation systems reflect that the systems themselves maintain sustainability. In addition, the reduction in lead time also shows why garment vendor companies trading with overseas buyers have used 3D virtual simulation systems in general.

However, their reactions also reflected that such advantages depend on the scale of the design companies and the levels of the designers’ digital skills. Participant 4 (a fashion designer and digital consulting company owner, 15 years of experience) mentioned the following:

I have always emphasized three key points for publicizing my company as follows: Using CLO3D, you can reduce lead time to produce garments, save the production cost, and protect environments. The best efficacy of 3D virtual simulation systems is that they can reduce lead time because the system can make decision-making faster. However, matters about costs and environments are case by case. The saved production cost can be offset by the increase in labor costs in small companies, whereas both cost savings and environmental protection can be accomplished in large companies, such as H&M.

In addition, the following statement of participant 3 (an individual designer brand entrepreneur, 6 years of experience) suggested that the level of digital designers’ skills is determined not only by their abilities to manipulate programs, such as CLO3D but also by their knowledge of various fields around garment production:

The most difficult problem for me in using CLO3D is that the system is time-consuming. Therefore, for me, using CLO3D efficiently does not always mean dealing with the program skillfully. I think that it also requires a larger scale of knowledge, including pattern-cutting and sewing methods because a variety of works, from designing garments in office, searching fabric stores, to handling factories are required to fashion designers.

The participants also noted that the significance of 3D virtual garment simulations emerged, especially under COVID-19. They demonstrated that COVID-19 has brought about the rise of interest in the system among companies and the demand for online platform consultation using the system. Participant 3 stated that COVID-19 has led to an increase in sales in his company.

### Discourses on the “uncanny valley” surrounding avatars

As an unpredicted finding, the majority of participants raised several troublesome issues around the use of avatars, especially in terms of their facial expressions and size accuracy. They noted that avatars of current 3D virtual simulation systems are not suitable for designing and retailing garments in industry because customers tend to prefer an ideal model face to their own ones, implemented with avatars. In this regard, participant 1 (a digital fashion designer and consultant, 8 years of experience) stated that “at this moment, 3D virtual simulation systems cannot perfectly embody an avatar’s face, nearly same as a user’s face. Actually, such reliability for materializing avatars has not been verified yet, and the level is somewhat low…”.

In fact, repulsions surrounding avatars of 3D virtual simulation systems also generated discourses on the “uncanny valley”. The concept was identified by Masahiro Mori, a professor in the field of robotics at the Tokyo Institute of Technology in 1970. In aesthetics, the “uncanny valley” (Mori, 2012[1970], p.1) is a hypothesized relationship between the degree of an object's resemblance to a human being and the emotional response to such an object. Along with the theory, the participants’ reactions indicated that the verisimilitude of avatars in 3D virtual simulation systems brought about cold and eerie feelings among them.

In this sense, participant 3 directly mentioned the term, “uncanny valley” during the interview as follows. “Although CLO3D is useful, basic avatars of CLO3D tend to make us feel slightly uncomfortable. Such an unavoidable “uncanny valley” seems to prevent garment images generated using CLO3D from being posted on online platforms for sales in reality.” In addition, he explained why garment products, including avatars that generate the “uncanny valley”, could not be immediately connected to sales on B2C (business-to-customer) online platforms and stated how his own company has dealt with such a problem. “Actually, when using CLO3D, there is a big difference in its image between including and excluding an avatar. So, creating digital contents, my company removes avatars as much as possible and instead, takes other methods, including replacing avatars with model scanning imagery and adding accessories to avatars”.

In this regards, participant 4 shared the point that garment vendor companies have usually used dummies instead of avatars, even in B2B (business-to- business):

There is a background in which garment vendors have used dummies rather than avatars to present their 3D garment designs. Because of the artificiality of avatars, there has been a great deal of requests for the verisimilitude of avatars, and several alternatives from live models’ scanned images to even celebrities’ faces have been offered … However, those alternatives failed to get good feedback … In fact, considering that the accuracy of garment sizes in B2B is one of the most important elements, neither avatars nor live model scanning images had lots of limitations and errors. However, because a dummy company, such as Alvanon, has produced dummies based on its own modeling information, the dummies could guarantee more exact fitting, reducing errors in sizes.

Participant 5 (a fashion designer of a garment vendor company, 4 years of experience) also shared his experience about buyers’ reactions to his 3D garment designs using CLO3D. “I have never gotten any complaints about fabrics or textures of 3D virtual simulating garments, but concerning the fitting, I have frequently received negative comments.”

Thus, the aforementioned discussions reveal that 3D virtual garment simulation using avatars has some difficulties in representing accurate sizes and brings about an “uncanny valley”, which has caused garment vendors to prefer dummies to avatars for B2B. In addition, the situations also indicate that it will still take more time for customers to accept 3D fashion garments in B2C markets. However, it infers that aesthetic and technical levels of verisimilitude have encouraged fashion designers to use 3D virtual simulation systems.

### Fashion design process with 3D virtual simulation systems

For the transition question about differences between conventional and digital design processes, using 3D virtual simulation systems, participants shared their common opinions, which focused on removing the stages of both flat drawing and toiling. In this regard, participant 3 stated that “after using CLO3D, the fashion design process in my company has changed slightly from the conventional design process. From the first stage, I start 3D toiling, based on prior designs from the company, and then I make 2D patterns, as opposed to the past design process. In this way, 3D toiling antecedes a 2D pattern.” Participant 5 also talked about some interplay between 2D pattern making and 3D toiling within 3D design works. “I remember that in the past, I continually repeated the process of pattern-making and toiling and then revised them again after designing a garment. However, I could leave out the toiling stage after using the 3D software.”

In the fashion design process, a basic design process has conventionally been shown as a conceptual idea: 2D sketch – 2D pattern – 3D toile – design/pattern alteration – sample (McQuillan, 2020). Meanwhile, the digital design process, using 3D virtual simulation systems, can transform the traditional process from 2D drawing to repeated modifications of 2D pattern making and 3D toiling simply into iterative interplays between 2D patterns and 3D simulations within the 3D software in a more sustainable way.

From the FGI interview, the author recognized that 3D design, rather than 2D design, has been gradually accustomed to designers in industry, and 3D virtual simulation systems have provided them with more convenient design process than conventional methods. Participant 4 described this process as follows. “Among designers, I have often seen cases in which they added 3D samples in the place of 2D flats to tech-packs to produce a prototype immediately. Because CLO3D is able to load 2D patterns and simulate them in 3D immediately, the patterns can be sent to manufacturing companies repeatedly. Therefore, although a designer has a short knowledge of patterns, he/she can create 3D samples easily.”

### 3D dynamic fashion design: Co-design and customization in online platforms

For the key questions with ten videos of 3D dynamic fashion garments, participants’ reactions showed various aspects around 3D dynamic fashion design, including expressive dimensions, entertainment effects, and customization possibilities. Three participants commented that expressive features of 3D dynamic fashion garments, which present various styles with transformable details and show fashion garments of changeable colors and graphical patterns, would be able to provide customers with various options in selecting their garments. More specifically, they revealed their interests in the traits of transforming from one to another design within a garment. Participant 1 stated “If a customer’s virtual garment is changeable in front of a FX mirror, it will look great! For example, when a customer is considering some kinds of colors or textile patterns of garments, or when a customer is stuck between designs with frills and without them, he/she will be able to have diverse options in selecting clothes, which will result in enhancing the customer’s satisfaction, as well.”

Participants also agreed with the opinion that 3D dynamic fashion garments could attract customers’ interests in a store, generating a public relations (PR) effect. Participant 2 (a digital fashion designer and consultant, 3 years of experience) noted that “certainly, I think that 3D dynamic fashion garments will be effective for catching customers’ attention. Although they are not always connected to customers’ purchases, 3D models or 3D fashion products posted on an online platform seem to promote interest in the brand.”

Most importantly, participants shared pros and cons around the possibility of co-design and customization from the ten given videos. In particular, sharing his own experience, which participated in a customized fashion project, called WITHIN24 + ALLSTUDIOS (n.d.), participant 3 described various aspects of garment customization, converged with ICT technology in detail. In collaboration with fifteen Korean young fashion brands, the project allowed consumers to choose their own styles when purchasing products. The digital technology used for this customized project included a 3D body scanner to measure customers’ body sizes, a customizing kiosk with CLO3D to enable customers to select various options, such as details, colors, patterns, and fabrics, and a virtual fitting system with an FX mirror to examine users’ sizes and perform their virtual fittings.

Noting some similarities between the case and the 3D dynamic fashion garments, he pointed out several issues about trying a custom-made fashion in an online platform for B2C, especially including costly and technical obstacles in building and maintaining an online platform. These issues also suggest the reason why the custom-made system is slightly difficult to apply to small-scale brands or companies:

For the project, I created two to three detail points as presets for a garment design to implement custom-made clothes. However, it was too expensive to build an online shopping platform, and the manpower consumption was also too high. As you know, I guess the 3D dynamic fashion garments you developed could also be very time-consuming because presenting rendered garment images or videos to customers took a lot of time. Time and cost issues are also related to designer staff proficiency.

In addition, participant 3 also indicated technical issues, such as a gap between 3D simulating garments and real garments. “Actually, the technology of FX mirror seems not to be sufficient at that moment. Although customers certainly feel interested in the customized technology at first, it looks difficult for virtual garments to provide customers with immediate assurance because there is still a slight gap between a garment rendered in CLO3D and a real garment.”

Participant 4 argued that above all, customers’ socio-cultural awareness to 3D virtual garments is too limited to accept customized clothes for B2C. She also added that the field of fashion design would be more difficult to implement customization than other fields, such as interior design, because it requires the most delicacy and accuracy. She noted that “the customized project, WITHIN24 + ALLSTUDIOS looked like too early an approach compared to its technical efficiency and audience awareness. In fact, in the case of a furniture brand, like Ikea, you can look at many digital products in online platform, whereas since clothes require very complicated mesh structures to simulate them, more delicate technical accuracy will be necessary for customers to accept them”.

From the FGI interview, the author could acknowledge that three issues need to be considered in the customization of 3D dynamic fashion garments; first, in a technical aspect the level of accuracy 3D virtual simulation systems can implement in size measurement and style expression, second, the issue of cost and time necessary for completing qualified 3D virtual garments, and finally, the audience awareness toward custom-made 3D garments. However, above all, the participants indicated that 3D dynamic fashion garments could have potential for customization, especially for B2B and even for B2C, as long as the technological level and socio-cultural reaction improve in the future.

### Future prospects of 3D virtual garments in the fashion and game industries

For the final questions, all the participants agreed that 3D virtual simulation systems would be necessary in the fashion industry in the near future. Participant 3 said that the current design method to draw flats using Illustrator on tech-packs will be definitely changed into 3D virtual simulations, which might be required for all staffs, including designers, pattern-makers, and samplers. He also predicted that fabric suppliers might provide designers with digital swatch data in place of real swatches.

In the future potential of 3D dynamic fashion garments, participants noted that they could have a possibility of mass customization. Two participants stated the prospect that the future fashion industry could be composed of diversified small-quantity products by mass customization. When the technical qualities of both avatars and virtual garments can perfectly implement reality, customers’ socio-cultural recognition of 3D custom-made garments will increase to accept them in an everyday situation, which will lead to the rise of online platforms for mass customization. Participant 3 stated, “Well… a customer could be scanned to produce his/her own avatar, and then with the data, including accurate sizes and design preferences, the customer could try on his/her personalized garment in an online platform”.

Participants also pointed out that 3D virtual fashion garments themselves, beyond just additional means for tangible garments, would be able to be connected to customers’ purchases. In this regard, participant 3 shared his ideas, stating a current phenomenon in which some luxury fashion brands have already started to sell virtual fashion garments for online game characters. “Just assume that my character put on garments in a game space. Then, I think that it will bring about customers’ demands to purchase garments for their characters and to wear their garments that are the same as their characters. In this way, such a collaboration between the fashion industry and the gaming industry will be able to create a positive synergy”. Participant 4 also suggested that the emergence of avatar models could more radically give us another possibility in online platforms. “There have already been some famous avatar models, and even digital modeling agencies. As they are considered entities in a virtual space in themselves, avatar models could be developed in much greater variety in the future in online fashion industries, like virtual models for look-books or music videos”.

Participants also emphasized that although digital technology, especially a 3D virtual simulation system, could suggest much potential to the fashion industry, comprehensive knowledge of physical garments, including patterns and textiles, as well as digital knowledge would still be important in industry.

## Discussion

In this section, the author returns to the design criteria suggested in the prior examples of dynamic fashion design (Table 1) to discuss the fertile ground of the creation of 3D dynamic fashion garments and digital fashion experts’ evaluations of them. This discussion provides future digital garments with several implications, which might be transferable to similar possible situations.

The criteria the author used to categorize previous cases were digital technology, dynamic range, wearability, expressivity, interactivity, sustainability, and context. The digital technologies used to develop 3D dynamic fashion garments is computer programs, including CLO3D and Aftereffects, and the dynamic range of 3D dynamic fashion garments is virtual.

First, the wearability of 3D dynamic fashion garments is only on screen, and the 3D dynamic fashion garments with realistic textures are fully portable, reacting avatar movements. As 3D virtual simulation systems allow fashion designers the immediacy of garment simulations without any physical sampling and as the fashion industry also begins to create digital-only clothing for their virtual models, dynamic fashion products, including 3D dynamic fashion garments will open up new opportunities for both designers and consumers in online platforms. However, 3D virtual simulation systems require more technological development, particularly including size accuracy, which poses future agenda to be solved for wearability of 3D dynamic fashion garments.

In fact, changing trends in the online fashion industry suggest that 3D dynamic fashion garments can be another possibility to be commercialized independently on online platforms rather than just supporting real garments. In this regard, Scolere (2019) noted that there have been self-promotion and self-presentation activities of creative professionals through social media platforms such as Instagram, Twitter, LinkedIn, and Pinterest. Gilliland (2020) also stated that there have been many online activities, from gaming-inspired fashion designs to a new category of virtual and shoppable styling games targeted at fashion consumers. Considering these aspects, some limitations of 3D dynamic fashion garments in wearability, which are only on screen will be able to be solved by collaborating with a variety of online industries and through technological developments.

Second, 3D dynamic fashion garments in expressivity will provide fashion designers with innumerable expressive ideas, presenting a fully multiple transformation in forms and styles, as well as surface changes of colors and textiles. In particular, 3D dynamic fashion garments represent plausible textual expressions of physical fabrics, rather than flat screen-based ones, overcoming the limits of prior dynamic garments. Moreover, they will also contribute to the demand of younger consumers, as they are highly interested in expressing their self-identities although online social networks.

In fact, various luxury brands, such as Marc Jacobs and Valentino, have become involved in games, such as Animal Crossing: New Horizons. In the game, players can often choose the appearance and style of the characters they play, and gamers can also compete with others or express themselves, differentiating online (Gilliland, 2020). In this regard, Mackey et al. (2019) claimed that by utilizing this type of dynamic fashion, fashion designers can expand the horizon of the wearers with a supplemental layer of meaning on top of the clothing through animated fabrics, engaging the wearer as the center of the fashion.

Meanwhile, avatars of the 3D dynamic fashion design brought discourses regarding the “uncanny valley”, which not only reflected Mori’s theory (2012 [1970]) that humanoid objects, which imperfectly resemble actual human beings, provoke uncanny or strangely familiar feelings of eeriness and revulsion in observers, but also proved that the concept has been rapidly applied to popular culture, with the increasing prevalence of AR/VR and 3D computer animations.

Third, in terms of interactivity, 3D virtual simulation systems, can allow customers to engage in the co-design process with their personalized avatars through online platforms, which shows great promise with mass customization for the fashion industry, such as online fashion stores, fashion promotions, and even entertainment. Potential users of 3D dynamic fashion garments might participate in the co-design process by asking 3D designers to design personalized avatars and garments. In a folder of personal digital devices or through an online platform, users might store their preferable styles as well as still or moving images(videos) as digital contents for a sort of customized virtual closet. Possibly within virtual spaces, users might enjoy 3D dynamic textiles or garments as much as traditional still textiles or garments.

DeRoulet (1993) defined mass customization as a feasible solution to meet customers’ needs more precisely while maintaining economies of scale by combining the best elements of mass production and customization. As a core component in the offerings of mass customizers, Wu (2010) stated that co-design is the process of involving consumers in co-creating a product, which combines individual consumers’ specifications with a company’s predesigned modules, and Fiore et al. (2001) defined apparel co-design as the process that a customer follows to choose an individualized combination of product style, fabric, color, and size from a finite group of options. In fact, customization and co-design have been recently on the rise in several brands, such as Adidas and Nike, as consumers are becoming increasingly demanding, time-driven, information-intensive, and highly individualistic (Sheth & Sisodia, 1997).

Customized online platforms equipped with 3D virtual simulation systems will support more various directions in aesthetics and functionality than under the current situation. As Ulrich et al. (2003) noted that advanced technologies, such as body scanning, CAD, and intelligent systems, enable customers to participate in the design of their own products and obtain a desirable fit, 3D virtual simulation systems will be able to greatly enhance the feasibility of consumer interaction and customization, meeting dynamic customers’ needs. In this aspect, 3D dynamic fashion garments might offer a kind of co-design and customization in presenting users predesigned modules of styles, design elements, and details.

Fourth, 3D dynamic fashion garments integrated into a base garment as a generic object will be directly connected to the sustainable practices of consumers, which could potentially be rechargeable and changeable without any waste in a virtual world. The sustainability of 3D virtual simulation systems also brings changes in the fashion design process and designers’ practices in the virtual and physical continuum, which interact with each other and will evolve with both on/offline contexts in multifaceted ways.

3D virtual simulation systems fundamentally maintain sustainable value because they enable designers to leave out prototype sampling and reduce the lead time in industry, which has an effect on the shift in the fashion design process. In this regard, McQuillan (2020) argued that the advantages of 3D software to augment the garment design process are clear, and this is particularly evident for zero-waste fashion design. In addition, since COVID-19, a great deal of interest in 3D virtual garment simulation systems has emerged among fashion companies, which pursue sustainable and digital-driven ideas. VOCAST (n.d.), which supports hundreds of brands by setting up their digital showrooms, claimed that the current COVID-19 pandemic might just have created the ideal circumstances for a long-awaited innovation boost, and fashion brands can take the opportunity to reimagine their lines within a digital-only space.

More significantly, a 3D dynamic fashion garment as an ultimate garment where a single garment functions as multiple garments (Choi, 2019a; Farrer, 2011; Mackey et al., 2017) also has the potential to lead to profitable sustainability in the future fashion industry. Introducing the concept of a base garment, Farren and Hutchison (2004) noted that the key reason the base garment concept is so important is because it allows an extension of that which people are already doing with fashion and garments, making choices about what they wear, how they appear, and what that appearance communicates to other people. Thus, 3D dynamic fashion garments add unique and sustainable value to the designed fashion offerings, contributing to the growth of online fashion platforms.

Finally, this study shows that online collaborations between interdisciplinary fields can generate new and innovative design outcomes for relevant themes. Social media platforms can also function as useful outlets to introduce and facilitate new communication modalities. In particular, digital technology integrated into the fashion design field will address the limitless potential to create highly versatile combinations in virtual worlds.

The context of 3D dynamic fashion garments can be considered fashion products in online platforms and entertainment devices which might be collaborated with fashion fields, beyond the usage of HCI research or digital users’ DIY (do-it-yourself). Their potential realms might cover customized fashion products in online stores, fashion promotions or advertisements, fashion film, gaming devices, and so on.

## Conclusions

This study introduced the term of 3D dynamic fashion garments to digital fashion design, which showed 3D moving visualizations with transformable styles and animated colors or textile patterns in a virtual space. The author presented the dynamic expressions and technologies programmed in 3D virtual simulation systems behind 3D dynamic fashion garments and explained how they bring new perspectives to the fashion design field, especially online fashion platforms.

This study has several limitations that suggest future studies. First, as the size of FGI participants for the survey is a bit limited, more numbers of participants will need to be interviewed for enhancing the validity of this study. Second, feedback on 3D dynamic fashion garments cannot only focus on fashion designers’ viewpoints but also center on consumers’ reactions and practices, which proposes future studies on comparing two groups or sub-groups within one group. Third, in developing 3D dynamic fashion garments, this study did not shed enough light on online collaborative process between designers in different fields. It also suggests future studies on the process of interdisciplinary online collaboration, or on other types of dynamic fashion design through the process. Finally, this study will be able to be extended toward a social media study on various cases of tangible or virtual dynamic fashion design.

## Supplementary Information

**Additional file 1.** Textile designs and 3D dynamic garment samples.

## Data Availability

The datasets generated and/or analyzed during the current study are available in the author’s google drive repository, which is linked to the figures, https://drive.google.com/drive/u/1/folders/1e0CoqjGX9WBiFb8pEuJR-3Y2iVnW5hVm.
