# Supplementary figures and images for: 3D dynamic fashion design development using digital technology and its potential in online platforms
Source: Fash Text. 2022 Mar 11;9(1):9. doi: 10.1186/s40691-021-00286-1 (PMC8914154; doi:10.1186/s40691-021-00286-1)

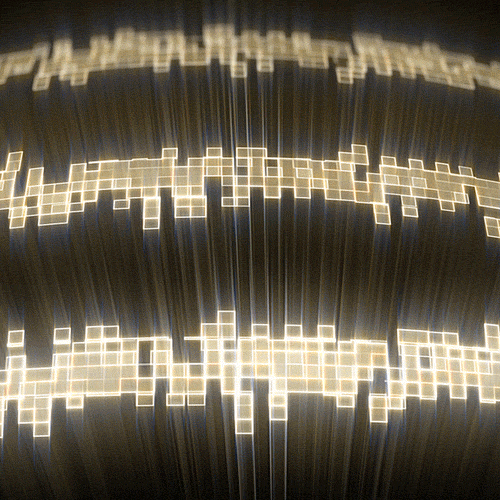

Supplement: Supplementary file 1 — Additional file 1. Textile designs and 3D dynamic garment samples. [file 40691_2021_286_MOESM1_ESM.zip › Figures (3D dynamic garments)/Figure4.Textile design2(sample4).gif]

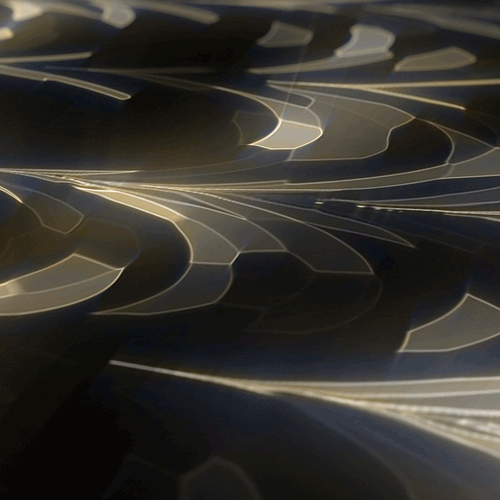

Supplement: Supplementary file 1 — Additional file 1. Textile designs and 3D dynamic garment samples. [file 40691_2021_286_MOESM1_ESM.zip › Figures (3D dynamic garments)/Figure4.Textile design1(sample4).gif]

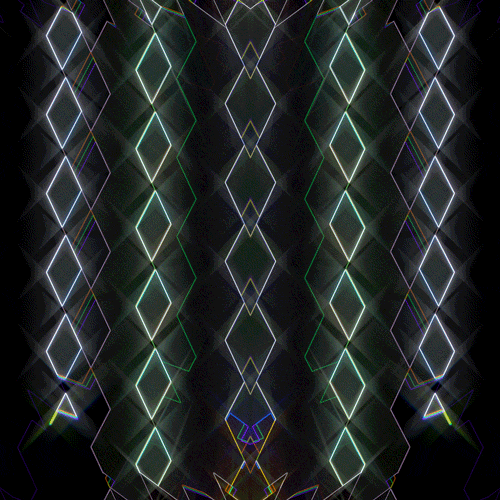

Supplement: Supplementary file 1 — Additional file 1. Textile designs and 3D dynamic garment samples. [file 40691_2021_286_MOESM1_ESM.zip › Figures (3D dynamic garments)/Figure3.Textile design2(sample3).gif]

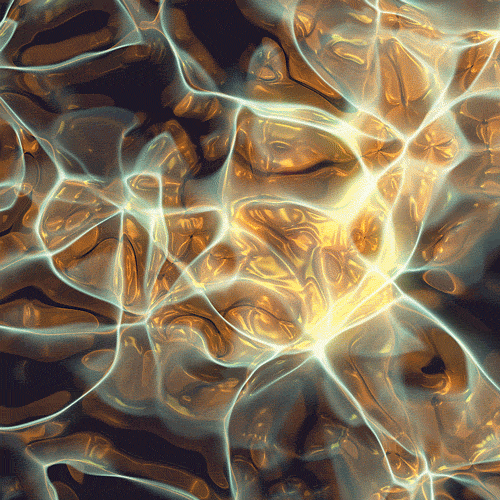

Supplement: Supplementary file 1 — Additional file 1. Textile designs and 3D dynamic garment samples. [file 40691_2021_286_MOESM1_ESM.zip › Figures (3D dynamic garments)/Figure1.Textile design(sample1).gif]

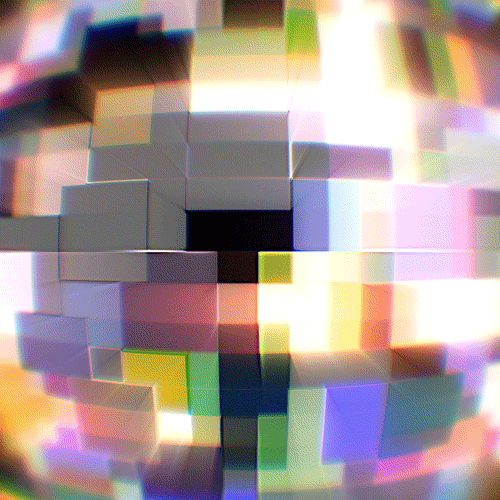

Supplement: Supplementary file 1 — Additional file 1. Textile designs and 3D dynamic garment samples. [file 40691_2021_286_MOESM1_ESM.zip › Figures (3D dynamic garments)/Figure2.Textile design1(sample2).gif]

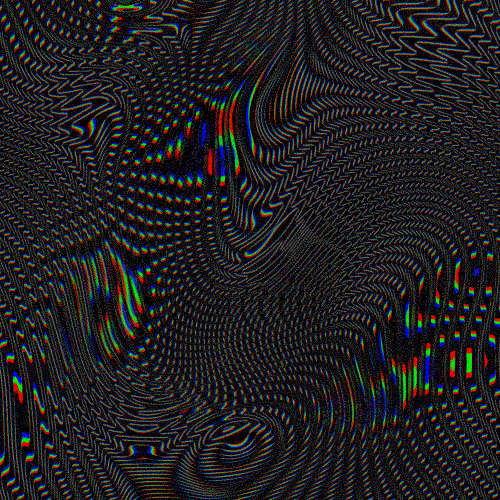

Supplement: Supplementary file 1 — Additional file 1. Textile designs and 3D dynamic garment samples. [file 40691_2021_286_MOESM1_ESM.zip › Figures (3D dynamic garments)/Figure3.Textile design1(sample3).gif]

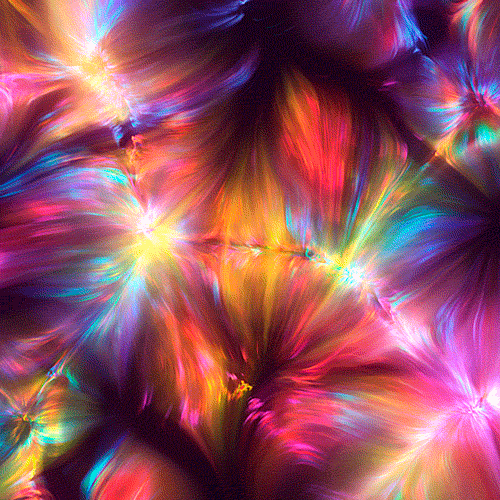

Supplement: Supplementary file 1 — Additional file 1. Textile designs and 3D dynamic garment samples. [file 40691_2021_286_MOESM1_ESM.zip › Figures (3D dynamic garments)/Figure2.Textile design2(sample2).gif]
